# Detection of male genital schistosomiasis (MGS) associated with human, zoonotic and hybrid schistosomes in Southern Malawi

**DOI:** 10.1186/s12879-024-09732-z

**Published:** 2024-08-19

**Authors:** Sekeleghe Kayuni, Lucas Cunningham, Bright Mainga, Dingase Kumwenda, David Lally Jnr, Priscilla Chammudzi, Donales Kapira, Gladys Namacha, Alice Chisale, Tereza Nchembe, Louis Kinley, Ephraim Chibwana, Bessie Ntaba, Gilbert Chapweteka, Waleke Khumalo, Henry Chibowa, Victor Kumfunda, Alexandra Juhasz, Sam Jones, John Archer, Angus M. O’Ferrall, Sarah Rollason, John Chiphwanya, Peter Makaula, E. James LaCourse, Janelisa Musaya, J. Russell Stothard

**Affiliations:** 1 Malawi Liverpool Wellcome Programme, Kamuzu University of Health Sciences, Queen Elizabeth Central Hospital, campus, 1 Chipatala Avenue, Private Bag 360, Blantyre 3, Chichiri, Malawi; 2 Department of Tropical Disease Biology, Liverpool School of Tropical Medicine, CTID Building, Pembroke Place, Liverpool, Merseyside, L3 5QA UK; 3 Pathology Department, School of Medicine and Oral Health, Mahatma Gandhi campus, Private Bag 360, Blantyre 3, Chichiri, Malawi; 4 Laboratory Department, Mangochi District Hospital, P.O. Box 52, Mangochi, Malawi; 5 Obstetrics and Gynaecology Department, Queen Elizabeth Central Hospital, 1 Chipatala Avenue, P.O. Box 95, Blantyre, Malawi; 6 Radiology Department, Queen Elizabeth Central Hospital, 1 Chipatala Avenue, P.O. Box 95, Blantyre, Malawi; 7 Nsanje District Hospital, Ministry of Health, Nsanje, Malawi; 8 Mangochi District Hospital, Ministry of Health, Mangochi, Malawi; 9 Institute of Medical Microbiology, Semmelweis University, Budapest, H-1089 Hungary; 10 School of Biosciences, The Sir Martin Evans Building, Cardiff University, Cardiff, CF10 3AX UK; 11 Community Health Sciences Unit (CHSU), National Schistosomiasis and Soil-Transmitted Helminths Control Programme, Ministry of Health, Area 3, Off Mtunthama Drive, Private Bag 65, Lilongwe, Malawi

**Keywords:** Lake Malawi, Shire River, Urogenital schistosomiasis, *Schistosoma haematobium*, *Schistosoma mattheei*, Semen

## Abstract

**Background:**

Male Genital Schistosomiasis (MGS) remains an often-overlooked chronic sequela of urogenital schistosomiasis in endemic areas of sub-Saharan Africa. As part of a 2-year longitudinal study on Hybridization of UroGenital Schistosomiasis (HUGS) in Malawi, a MGS sub-study was conducted to assess whether hybrid schistosomes were incriminated.

**Methods:**

During recruitment, demographic, health and socio-economic data were collected through individual questionnaire interviews in Mthawira community from Nsanje District along Shire River and Samama community from Mangochi District along Lake Malawi shoreline. Urine and semen samples were collected and analysed to determine the identity of schistosome infection. Urine filtration and microscopy, direct microscopy of semen and its sediments (after centrifugation) were performed. Thereafter, the sediments were examined by molecular DNA analysis with a novel two-tube real-time PCR assay. The participants were also screened for Human papilloma virus (HPV) and other sexually transmitted infections (STIs).

**Results:**

Twenty-two men were recruited for the sub-study, 8 in Nsanje District and 14 in Mangochi District, with a median age of 22.0 years. By microscopy, ten (45.7%) participants had *Schistosoma* ova in their urine, 11 (50.0%) in semen while 16 (72.7%) were positive by real-time PCR. One participant had both *S. haematobium* and *S. mattheei* ova in his semen, three showed symptoms, and one had a mixed infection of *S. mansoni* and possible *S. haematobium*-*S. mattheei* hybrid. Twelve men had detectable high-risk HPV serotypes 16, 18 and others while six had *Trichomonas vaginalis* and other STIs.

**Conclusion:**

Zoonotic and hybrid schistosomes can cause MGS similar to human schistosomes, which can be co-infected with HPV and STIs, thereby posing a new challenge in diagnosis, management and control measures in resource poor settings. Increased awareness of these infections among local communities and primary healthcare workers and improvement of disease management are needed and advocated.

**Supplementary Information:**

The online version contains supplementary material available at 10.1186/s12879-024-09732-z.

## Background

Schistosomiasis remains the most common snail, freshwater-borne neglected tropical disease (NTD) affecting over 224 million people [1].The genital form of this disease, a sequela of a chronic infection caused primarily by *Schistosoma haematobium*, remains overlooked and understudied [2].

Male genital schistosomiasis (MGS) was first described in a 14-year old Egyptian boy with an enlarged scrotum who presented with epidydimal schistosomiasis and in an English soldier who complained of haemospermia (blood in semen) concurrently with urinary schistosomiasis. The descriptions were made in 1911 at Kasr-el-Ainy Hospital in Egypt by Professor Madden [3]. MGS typically develops during the early stages of life and later becomes symptomatic as the disease progresses over time, causing spontaneous pelvic pain, during coitus and/or upon ejaculation. Other symptoms may include changes in the ejaculate, erectile dysfunction or discomfort and infertility [4–6].

Although observations suggest that genital organs are commonly affected by *S*. *haematobium* ova, and to a lesser extent with *S*. *mansoni*, the full extent of morbidity associated with MGS in endemic areas remains poorly understood. This is most clearly demonstrated by autopsy studies and case reports [7]. Other schistosomes of medical importance such as *S. intercalatum* and *S. guineensis* as well as those that infect animals, *S. mattheei*,* S. bovis* and *S. curassoni* have also infected humans [8]. In addition to the new endemic areas with local foci of infection such as Corsica, France [9, 10], there is increasing evidence of hybridization events between human and animal schistosome species [11–13], which pose an additional challenge to disease control.

Like Female Genital Schistosomiasis (FGS), MGS has often been undiagnosed and misdiagnosed as a sexually transmitted infection (STI) due to the similar symptoms and technical diagnostic challenges in rural and resource-limited endemic areas [14]. Furthermore, the association between zoonotic and hybrid infections in the context of MGS has not been fully investigated. Also, zoonotic and hybrid schistosomes are increasingly becoming recognized as an emerging public health problem [11, 12].

Certainly, MGS have been observed to increase the risk of human immunodeficiency virus (HIV) transmission in men from endemic areas of sub-Saharan Africa (SSA), through increased viral shedding in genital fluids and inflammatory cells as well as immunological mediators enhancing transmission [15, 16]. Therefore, improving the diagnosis, treatment and management of MGS with praziquantel (PZQ) could aid the control of schistosomiasis and HIV, for which overlapping prevalences have been observed in these endemic areas, particularly in SSA [17, 18].

As part of a larger community-based study funded by National Institute for Health Research (NIHR) and Wellcome Trust UK, entitled ‘Hybridization in UroGenital Schistosomiasis (HUGS)’ comprising of human, animal and snail vector surveys, two districts of Nsanje and Mangochi in Southern Malawi were selected. 1,065 males were recruited in the study at baseline in June and July 2022, out of a total of 2,271 individuals across both districts. An MGS sub-study was conducted during the first annual human follow-up survey in June and July 2023 to assess the presentation of MGS associated with human, zoonotic and hybrid schistosome species among recruited males, thereby characterising infections of their genital tract in greater detail.

## Methods

### Study area, design and participants

**Fig. 1:**
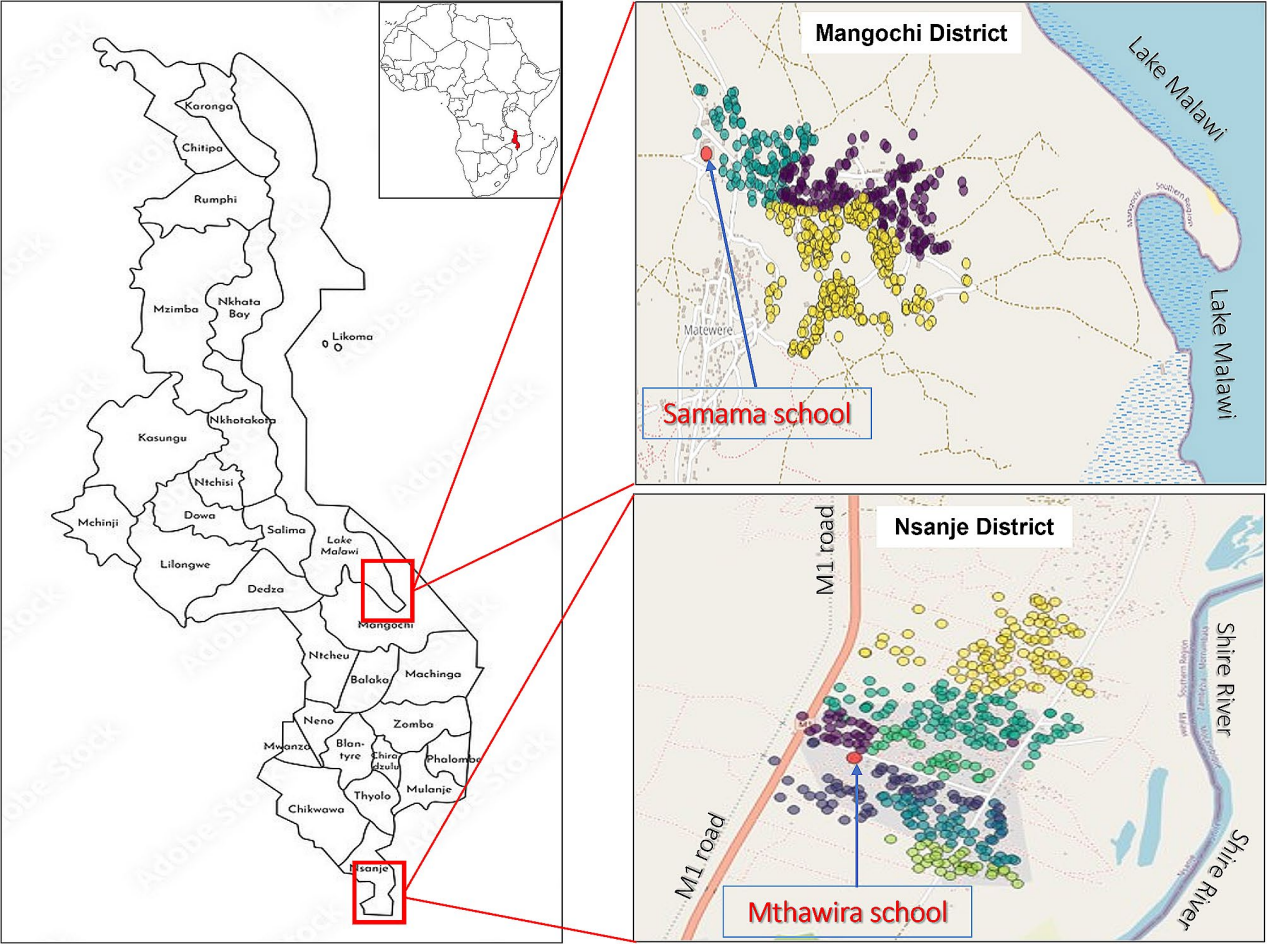
Map showing two study communities around Samama School in Mangochi District and Mthawira School in Nsanje District of Southern Malawi where participates came from

As part of the annual Follow up survey for the main HUGS human study in Mthawira community from Nsanje District along Shire River and Samama community from Mangochi District on southern shoreline of Lake Malawi (Fig. 1) were selected to recruit men aged 18 and above who had active egg-patent schistosomiasis at baseline in 2022. These individuals were invited to participate in the MGS sub-study. Written informed consent was sought after adequate awareness before enrolment into the study.

### Data collection

Demographic data were collected using individual questionnaire interviews to gather information about their health, socio-economic status, water contact behaviours, availability of livestock and MGS related symptoms (refer to Supplementary file).

### Urine and semen microscopy

Urine sample was submitted in a 120 ml clear container during the study visit and underwent filtration and microscopical examination to detect the presence of *Schistosoma* ova in 10 ml of well-mixed sample [19]. Additionally, reagent dipstick and point-of-care circulating cathodic antigen (POC-CCA) testing were performed on each sample. They were then asked to submit a semen sample into a clear plastic bag for direct microscopy in the field, before being centrifuged and repeat microscopy of the sediments. The sediments were then added to 1 ml of 70% ethanol in preparation for molecular analyses at Liverpool School of Tropical Medicine (LSTM), United Kingdom.

## Molecular analyses

### High-resolution melt (HRM) real-time PCR

The semen samples were subjected to DNA extraction using the DNAmini blood and tissue kit (Qiagen) according to the manufacturer’s instructions, with the addition of a bead-beating treatment using approximately 0.5 g of 1.4 mm ceramic beads per-sample before incubation with the ATL/Proteinase K. Phocine herpes virus was added along with addition of the first buffer, to serve as an internal extraction control. An empty tube was included alongside the samples as a negative extraction control.

Real-time molecular PCR analysis was performed using a novel two-tube high-resolution melting (HRM) real-time PCR assay targeting both the nuclear (nDNA) and mitochondrial (mtDNA) genomes of the following six species, viz. *S. mattheei*,* S. curassoni*, *S. bovis*,* S. haematobium*, *S. mansoni* and *S. margrebowiei*. The mtDNA real-time PCR consists of six species-specific primer pairs in a multiplex, while the nDNA real-time PCR targets two conserved regions of the ITS2 gene flanking a variable region accessible to species differentiation. Comparing the melting temperature for the real-time PCR product of each assay allows for species-identification.

In an HRM assay, the most important diagnostic characteristic is the melting temperature of the PCR product generated for the various target species for both the mitochondrial and nuclear real-time PCR reactions. The HRM is based on the melting of double stranded DNA. The exact temperature at which half of the DNA strands are in the single-stranded state is called the melting temperature (Tm). To optimise cycling conditions, a comparison of the Ct values at three annealing temperatures (58⁰C, 59⁰C and 60⁰C) was carried out.

A DNA library containing the six target species was curated to properly validate and test the novel HRM two-tube real-time PCR assay. The oligos designed during the HUGS study were screened using the HRM ITS2 assay and the mtDNA species-specific HRM assay as indicated above, in 12µL reactions consisting of 6µL of Type-it HRM Supermix (Qiagen), 400nM primers and 2µL template, with remaining volume consisting of nuclease free water.

### TaqMan real-time PCR

#### Schistosome real-time PCR

The two probe-based reactions included a duplex reaction using the generic ITS1 schistosome primer/probe set and the primer-probes for PhHv internal control detection. The second probe-based reaction was a triplex reaction consisting of the ribosomal 16 S species-specific primer-probe sets for *S. haematobium* and *S. mansoni* and the PhHv primer-probe set. All real-time PCR assays were performed for 40 cycles. Probe-based reactions included similar volumes and concentrations of appPROBE No ROX supermix (Appleton Woods), primer and DNA template with the addition of 100nM of reaction-specific probes.

#### HPV real-time PCR

Alongside the screening for *Schistosoma spp*, HPV markers were also screened for using the QIAscreen HPV PCR Test kit (Qiagen, Manchester UK). This kit is capable of screening for two high-risk genotypes, 16 and 18 alongside the following, lower risk, types: 16, 18, 31, 33, 35, 39, 45, 51, 52, 56, 58, 59, 66, 67 and 68.

### Semen analysis for STIs

In addition to the HPV screening, the seminal sediments were also analyzed for other STIs using the EasyScreen™ STI Kit (by SydPath, Sydney, Australia). This kit simultaneously detected the 12 common STIs: *Chlamydia trachomatis*, *Neisseria gonorrhoeae*, *Lymphogranuloma venereum* (LGV), *Mycoplasma genitalium*, *Trichomonas vaginalis*, *Ureaplasma* spp., *Candida* spp., *Mycoplasma hominis*, *Streptococcus agalactiae* (Group B *Streptococcus*), *Treponema pallidum*, Herpes Simplex Virus 1 (HSV1) and Herpes Simplex Virus 2 (HSV2).

### Data analyses

The results of the diagnostic tests were analysed using non-parametric tests due to the small sample size and non-normal distribution in the Excell program. The results were tabulated and presented accordingly.

### Ethical considerations

Ethical approval for the study was granted by the College of Medicine Research Ethics Committee (COMREC), Kamuzu University of Health Sciences (KUHeS), Malawi, (Approval number: P.08/21/3381) and the LSTM Research Ethics Committee (LSTM REC) in the United Kingdom (registration number: 22–028). Informed consent to participate in the study was obtained from all of the participants. Privacy and confidentiality were maintained throughout the study.

## Results

Twenty-two male participants were recruited into the MGS sub-study, 8 from Nsanje District and 14 Mangochi District. The median age for the participants was 22.0 years (range: 18–39), 25.5 years in Nsanje (range: 19–39) and 20.0 in Mangochi (range: 18–32) (Table 1).

Three participants reported symptoms associated with MGS, namely blood in semen (haemospermia), sores and pain in the genitalia, pain during coitus and ejaculation. Only one participant (J) with symptoms had *Schistosoma* ova in their urine and semen.

**Table 1:** Demography, MGS symptoms and laboratory findings of the participants

Participant ID	Age (years)	MGS-associated Symptoms	Urine filtration egg count	Semen microscopy		
				S. h. eggs	S. matt. eggs	Observations
A	19	None	1–9	8	1	Normal
B	18	None	50+	25	0	Normal
C	18	None	10–49	6	0	Haemospermia (blood in semen), loose consistency, azoospermia
D	18	None	0	15	0	Colour change, loose consistency, azoospermia
E	21	None	0	2	0	Normal
F	28	None	0	0	0	Normal
G	27	None	1–9	1	0	Normal
H	19	None	0	5	0	Normal
I	30	None	1–9	0	0	Normal
J	22	Blood in semen (haemospermia), pain on coitus, ejaculation, in genitalia, sores	10–49	9	0	Normal
K	18	None	0	0	0	Normal
L	21	Pain and sores in the genitalia	0	0	0	Normal
M	18	None	10–49	100	0	Haemospermia, loose consistency, dead spermatozoa
N	32	None	1–9	0	0	Normal
O	32	Pain on coitus, ejaculation, sores on the genitalia	0	0	0	Normal
P	39	None	0	0	0	Normal
Q	30	None	0	0	0	Normal
R	26	None	0	0	0	Normal
S	19	None	0	1	0	Normal
T	22	None	0	0	0	Normal
U	22	None	1–9	500	0	Normal
V	25	None	1–9	0	0	Normal

Note: *S. h. = Schistosoma haematobium*, *S. matt.* = *Schistosoma mattheei*

**Fig. 2:**
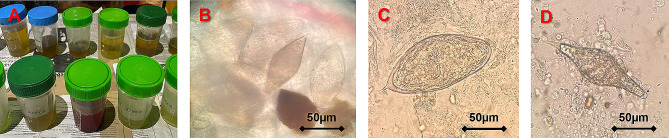
Schistosome eggs observed in samples submitted by the study participants: **2A** shows urine samples; **2B** shows multiple *Schistosoma haematobium* eggs and one odd *Schistosoma* egg in urine samples; **2C** shows *Schistosoma haematobium* egg observed in semen sample; and **2D** shows *Schistosoma mattheei* egg in semen sample. (*Photo images courtesy of Professor JR Stothard*,* Dr Sekeleghe Kayuni and Mr Bright Mainga*)

Ten (45.7%) participants had *Schistosoma* ova in their urine, two (25.0%) in Nsanje and 8 (57.1%) in Mangochi (Fig. 2). Eleven (50.0%) participants had *Schistosoma* ova detected on semen microscopy, two (25.0%) in Nsanje and 9 (64.3%) in Mangochi. One participant, A, (19 yo) had both *S. haematobium* and *S. mattheei* ova, two participants, C and D, (both 18 yo) had haemospermia, loose semen consistency and azoospermia, while one participant, M, (18 yo), also had dead spermatozoa. Of note, 14 (63.6%) participants had *Schistosoma* ova in their urine or semen, of which three (45.5%) had the ova in urine only, four in semen only and seven in both urine and semen.

Using real time PCR, 16 (72.7%) participants were positive for *Schistosoma* infection, four (50.0%, *n* = 8) in Nsanje and 12 (85.7%, *n* = 14) in Mangochi (Table 2). Of these, three (participants P, S, U) had only *S. haematobium* detected, one with only *S. mattheei* (V), three with mixed *S. haematobium* and *S. mattheei* infections (A, C, G), two with mixed *S. haematobium* and *S. mansoni* infections (B, N) and one with mixed infection of *S. mansoni* and possible *Schistosoma haematobium*-*Schistosoma mattheei* hybrid (F).

**Table 2:** Results of molecular analysis using real-time PCR of the participants’ semen

Participant ID	Age (years)	Urine filtration egg count	Semen microscopy		Cq Gen	Cq Mito	HRM Tm1(°c)	HRM Tm2(°c)
			S. h. eggs	S. matt. eggs				
A	19	1–9	8	1	16.8	27.3	71.2	0.0
B	18	50+	25	0	18.5	28.9	76.3	0.0
C	18	10–49	6	0	16.5	31.2	71.5	0.0
D	18	0	15	0	20.4	0.0	0.0	0.0
E	21	0	2	0	0.0	0.0	0.0	0.0
F	28	0	0	0	24.1	0.0	71.4	76.2
G	27	1–9	1	0	22.4	28.7	71.4	0.0
H	19	0	5	0	25.3	0.0	0.0	0.0
I	30	1–9	0	0	23.6	0.0	0.0	0.0
J	22	10–49	9	0	25.5	0.0	0.0	0.0
K	18	0	0	0	0.0	0.0	0.0	0.0
L	21	0	0	0	37.8	0.0	0.0	0.0
M	18	10–49	100	0	20.4	0.0	0.0	0.0
N	32	1–9	0	0	23.7	25.4	76.1	0.0
O	32	0	0	0	0.0	0.0	0.0	0.0
P	39	0	0	0	36.6	0.0	0.0	0.0
Q	30	0	0	0	0.0	0.0	0.0	0.0
R	26	0	0	0	0.0	0.0	0.0	0.0
S	19	0	1	0	28.7	0.0	0.0	0.0
T	22	0	0	0	0.0	0.0	0.0	0.0
U	22	1–9	500	0	21.6	0.0	0.0	0.0
V	25	1–9	0	0	0.0	32.3	71.9	0.0

Note: Cq = Threshold cycle, *S. h. = Schistosoma haematobium*, *S. matt.* = *Schistosoma mattheeii*, HRM = High-Resolution Melt, Tm = melting temperature, C = degrees Celsius

Testing for HPV showed 12 (54.5%) participants had detectable serotypes associated with cervical and penile carcinoma, four (22.7%) with type 16, two with type 18 while 10 (45.5%) had any other serotypes detected (Table 2). Of these 12 with detectable HPV, all except one had detectable *Schistosoma* on real time PCR and 7 had ova in semen.

**Table 3:** Results of the human papilloma virus (HPV) serotypes real time PCR

Participant ID	Age	Microscopy		Cq Gen	Cq Mito	HRM Tm1(°c)	HRM Tm2(°c)	HPV16	HPV18	Any HPV
		S.h.	S. matt.							
A	19	8	1	16.8	27.3	71.2	0.0	34.0	0.0	36.8
B	18	25	0	18.5	28.9	76.3	0.0	0.0	0.0	24.9
C	18	6	0	16.5	31.2	71.5	0.0	0.0	0.0	36.9
D	18	15	0	20.4	0.0	0.0	0.0	0.0	26.3	0.0
E	21	2	0	0.0	0.0	0.0	0.0	0.0	0.0	33.9
F	28	0	0	24.1	0.0	71.4	76.2	19.6	0.0	19.7
G	27	1	0	22.4	28.7	71.4	0.0	0.0	19.8	19.0
H	19	5	0	25.3	0.0	0.0	0.0	0.0	0.0	21.4
I	30	0	0	23.6	0.0	0.0	0.0	18.1	0.0	19.9
L	21	0	0	37.8	0.0	0.0	0.0	0.0	0.0	30.3
N	32	0	0	23.7	25.4	76.1	0.0	27.1	0.0	27.2
P	39	0	0	36.6	0.0	0.0	0.0	0.0	0.0	37.5

Note: HPV = Human Papilloma Virus

The participants were also screened for other STIs and six participants had detectable *T. vaginalis* while 2 had other STI in their semen (I - *Candida* spp., *Mycoplasma hominis*, Herpes simplex virus (HSV) type 1; N - *Trichomonas vaginalis*,* Chlamydia trachomatis*,* Ureaplasma* spp., *Candida* spp., *M. hominis*, HSV types 1 and 2).

**Table 4:** Real-time PCR results on the sexually transmitted infections (STIs) screen

Participant ID	Age	Urine filtration	Microscopy		Cq Gen	Cq Mito	HRM Tm1(°c)	HRM Tm2(°c)	HPV	TV	Other STIs
			S. h. Eggs	S. matt. eggs							
I	30	1–9	0	0	23.6	0.0	0.0	0.0	+	29.3	+^α^
K	18	0	0	0	0.0	0.0	0.0	0.0	-	34.1	-
L	21	0	0	0	37.8	0.0	0.0	0.0	+	34.5	-
M	18	10–49	100	0	20.4	0.0	0.0	0.0	-	35.1	-
N	32	1–9	0	0	23.7	25.4	76.1	0.0	+	23.3	+^β^
P	39	0	0	0	36.6	0.0	0.0	0.0	-	37.5	-

Note: HPV = Human Papilloma Virus, TV = *Trichomonas vaginalis*, STIs = Sexually Transmitted Infections, ^α^*Candida* spp., *Mycoplasma hominis*, Herpes simplex virus (HSV) type 1, ^β^*Trichomonas vaginalis*,* Chlamydia trachomatis*,* Ureaplasma* spp., *Candida* spp., *M. hominis*, HSV types 1 and 2

## Discussion

Male genital schistosomiasis is seldomly recognised and frequently undiagnosed and underappreciated in endemic areas, despite being as commonly prevalent as urogenital schistosomiasis. Our cohort of 22 men showed a significant prevalence of MGS, *Schistosoma* ova was detected in semen of eleven men (50.0%) while sixteen men (72.7%) were positive on real-time PCR for *Schistosoma* infection. Previous studies have shown that prevalence of MGS in men along south Lake Malawi was 10.4% and 26.6% by semen microscopy and real-time PCR respectively and in Madagascar, it was 43% in semen samples with elevated eosinophil cationic protein (ECP) [6, 20, 21].

To our knowledge, this is the first prospective, longitudinal study to examine the MGS burden in context of non-human schistosomes including zoonotic and hybrid types. The extent of the morbidity resulting from these zoonotic and hybrid MGS infections remains poorly understood although these schistosomes in SSA are increasingly becoming recognized as an emerging public health problem [11, 12].

[6, 20] Interestingly, we detected zoonotic *S. mattheei* ova along with human *S. haematobium* in the semen of a participant A, a novel scenario never before observed in MGS infected people. Proximity to domesticated livestock such as goats, sheep and cattle could be associated with the zoonotic infection.

Furthermore, the use of our novel two-tube real-time PCR, resulted in the detection of more participants with MGS. Some participants also had mixed infections with zoonotic *S. mattheei* along with human *S. haematobium* and *S. mansoni* with one possibly having a mixed *S. haematobium*-*S. mattheei* hybrid and *S. mansoni* infection. Such a triple infection is groundbreaking finding among people with MGS in endemic areas and demonstrates the importance of advanced molecular diagnostics in such diseases and need to implement One Health measures to control and elimination of schistosomiasis as public health problem in endemic areas [11].

Although some participants reported symptoms associated with MGS, namely haemospermia, changes in semen colour and consistency and azoospermia which have been reported in other studies, the majority of participants reported no symptoms. Since the symptoms may be due to some STIs, we also performed HPV screening. The result was that 15 participants tested positive for a range of HPV types, seven of which were high risk types 16 (*n* = 5) and 18 (*n* = 2). The presence of multiple MGS, HPV and STIs co-infections in these participants may accelerate the severity of each disease progression and thus adversely impact the health wellbeing of affected men in the community. This then highlights the importance of a combination of history taking and medical examination by well-trained health workers as well as improved relevant laboratory and diagnostic testing in rural and resource-limited settings in endemic areas [14, 22].

The study was limited in the low number of study participants due to the sensitivity of the condition as well as the poor health seeking behaviour of men in most endemic areas. For a wider context, there’s need for further, larger stratified studies in Malawi and other endemic areas to provide a representative picture of the MGS outcome.

## Conclusions

Here in Malawi, human, zoonotic and hybrid schistosomes can cause MGS. Likewise, people with MGS may be co-infected with HPV and STIs, posing a further challenge in diagnosis, treatment, management and control interventions in resource poor settings. There is a clear imperative to raise awareness of MGS among primary healthcare workers and to encourage the National Control Program to adequately address MGS.

### Electronic supplementary material

Below is the link to the electronic supplementary material.

Supplementary Material 1

## Data Availability

The datasets generated and analyzed for this study can be made available by contacting the corresponding author.
