# Supplementary material for: Detection of male genital schistosomiasis (MGS) associated with human, zoonotic and hybrid schistosomes in Southern Malawi
Source: BMC Infect Dis. 2024 Aug 19;24:839. doi: 10.1186/s12879-024-09732-z (PMC11331596; doi:10.1186/s12879-024-09732-z)
Supplement: Supplementary file 1 — Supplementary Material 1 [file 12879_2024_9732_MOESM1_ESM.pdf]

5.1 Participant's Questionnaire (English)

Hybridization in urogenital schistosomiasis (HUGS): A multidisciplinary longitudinal population study revealing the transmission biology, epidemiological impact and clinical importance of *Schistosoma haematobium*-hybrids in Malawi.

A Structured Knowledge Attitudes and Practice (KAP) Survey Questionnaire

Introduction: The HUGS Project, would like to request for your kind cooperation to participate in this survey in order to collect information from you to assess the level of knowledge, attitudes and practices related to schistosomiasis in this community.

Enumerator ID: /_/_/_/_/

Information and procedure for eliciting informed written consent

[**Enumerator:** Please verify if the participant has agreed to take part in the study and has provided a written informed consent before proceeding with the survey].

1. Have you read out the project information sheet and a written informed consent has been granted by the respondent or his/her parent/guardian? **1** = Yes (Proceed with the interview); **2** = No (Do not proceed with the interview, request for informed written consent)

Instructions: Please indicate the responses by choosing the corresponding codes. Where the options are **correct** or **incorrect**, please refer to the guiding notes at the end of this questionnaire.

Part A: Geographical information

2. Districts: **1** = Mangochi; **2** = Nsanje
3. Nearest Health Centres: ....
4. Villages: ....

Part B: Socio-demographic information for respondent

5. Respondent ID number: ***/-/-/-/***
6. Age: ***/-/-/*** = (## years; **00** = Do not know or remember)
7. Sex: **1** = Male; **2** = Female
8. Marital status: **1** = Single; **2** = Married; **3** = Divorced; **4** = Widowed; **5** = Separated; **6** = Other

9. Number of people in the household: Total = ***/-/-/***; [Male = ***/-/-/***; Female = ***/-/-/***;] [Adults (> 18 years) = /-/-/; Young (< 18 years) = /-/-/]
10. Tribe: **1** = Chewa; **2** = Lomwe; **3** = Mang'anja; **4** = Ngonde; **5** = Ngoni; **6** = Sena; **7** = Tonga; **8** = Tumbuka; **9** = Yao; **0** = Other
11. Religion: **1** = Atheist; **2** = Christian; **3** = Moslem; **4** = Traditional; **5** = Other
12. Highest education level: **1** = Primary; **2** = Secondary; **3** = Tertiary; **4** = None
13. Occupation: **1** = Business; 2 = Farmer; **3** = Fisher; **4** = Employed; **5** = Schooling; **6** = Unemployed; **7** = Other

Part C: Knowledge, attitudes and practices about schistosomiasis

14. Do you know what schistosomiasis is? **1** = Yes; **2** = No
15. If yes, please tell me what schistosomiasis is? **1** = Correct; **2** = Incorrect
16. Do you know what causes schistosomiasis? **1** = Yes; **2** = No
17. If yes, what is it that causes schistosomiasis? **1** = Correct; 2 = Incorrect
18. How many types of schistosomiasis do you know?
    1 = One type; **2** = Two types; **3** = More types; **4** = Do not know
19. Do you know the signs or symptoms of schistosomiasis? **1** = Yes; **2** = No
20. If yes, please mention any sign or symptoms of schistosomiasis you know? **1** = Correct; **2** = Incorrect
21. Which organs in the body does the schistosomiasis parasites attack? **1** = Bladder only; **2** = Abdomen only; 3 = Bladder and abdomen; **4** = Other organs; **5** = Do not know
22. Do you know how healthy people get infected with schistosomiasis? **1** = Yes; **2** = No
23. If yes, explain to me how a healthy person can get infected with schistosomiasis? **1** = Correct; **2** = Incorrect
24. Do you know how schistosomiasis is transmitted from a sick person to a healthy person? **1** = Yes; **2** = No
25. If yes, explain to me how schistosomiasis is transmitted from a sick person to a healthy person? **1** = Correct; **2** = Incorrect
26. Do you know what organism acts as intermediate host for schistosomiasis parasites? **1** = Yes; **2** = No, **3** = Do not know
27. If yes, what organism acts as an intermediate host for schistosomiasis parasites? **1** = Snails; **2** = Others; **3** = Do not know
28. Do you know how one can prevent contracting schistosomiasis? **1** = Yes; **2** = No
29. If yes, how can one prevent contracting schistosomiasis? **1** = Correct; **2** = Incorrect

30. Is schistosomiasis treatable by drugs? **1** = Yes; **2** = No
31. Do you know what problem(s) schistosomiasis can cause to a person if not treated? **1** = Yes; **2** = No
32. If yes, mention any example of problems that schistosomiasis can cause to a person if not treated? **1** = Correct; **2** = Incorrect

Are you experiencing the following symptoms now? In the last month? Kindly indicate your agreement with the following statements by ticking **(√)** in the appropriate corresponding box:

**For any participant**						
	**Symptom**	**Currently**	**In last month**	**Not in last month**	**Do not remember**	**Refused**
33.	Fever					
34.	Headache					
35.	Fatigue					
36.	Abdominal cramping (pain)					
37.	Pain during urination (dysuria)					
38.	Frequency of urination					
39.	Colour of urine					
40.	Blood in urine (haematuria)					
41.	Blood in stool					
42.	Other (please specify):					

**For female participants only**						
	**Symptom**	**Currently**	**In last month**	**Not in last month**	**Do not remember**	**Refused**
43.	Abnormal vaginal discharge					
44.	Pain or discomfort during coitus					
45.	Vaginal bleeding after coitus					
46.	Afraid that you might have pain or bleeding after coitus					
47.	Sores on your private parts					
48.	Other (please specify):					

For male participants only						
	**Symptom**	**Currently**	**In last month**	**Not in last month**	**Do not remember**	**Refused**
49.	Blood in semen					
50.	Pains during and / or after coitus					
51.	Pains on ejaculation					
52.	Pains of your private parts					
53.	Sores on your private parts					
54.	Other (please specify):					

55. Have you ever suffered from schistosomiasis? **1** = Yes; **2** = No
56. If yes, did you get drugs for treatment of schistosomiasis? **1** = Yes; **2** = No
57. Have you recently received drugs for schistosomiasis? **1** = Yes; **2** = No
58. If yes, when did you recently receive the drugs for schistosomiasis?
    1 = Within past one week; **2** = Within a month ago; **3** = Within one year ago; **4** = Over one year ago; **5** = Do not remember
59. Where did you get the drugs from?
    1 = Health Facility; **2** = In Community; **3** = Store or grocery; **4** = School; **5** = Other
60. Who dispensed the schistosomiasis drugs to you?
    1 = Facility health worker; **2** = Community health worker; **3** = Community volunteer; **4** = Other
61. Did you experience any problem(s) after taking schistosomiasis drugs? **1** = Yes; **2** = No
62. If yes, what problem did you experience after taking schistosomiasis drugs?
    1 = Drowsiness/dizziness; **2** = Nausea/vomiting; **3** = Abdominal pain; **4** = Fever/sweating **5** = Allergy/rash; **6** = Others
63. How many other members of your household received drugs for schistosomiasis within the past year? ***/-/-/***
64. Are schistosomiasis drugs readily accessible in this village? **1** = Yes; **2** = No
65. What is the closest you have ever lived to a source of fresh water (river or lake)
    1. <5 minutes' walk; **2.** 5-10 minutes' walk; **3.** 11-20 minutes' walk;
    4. 21- 30 minutes' walk; **5.** more than 30 minutes' walk
66. How long did you live close to a source of fresh water?
    1. I have never lived closer than a 1 hour walk to fresh water; **2.** <1 month; **3.** 1-3 months;
    4. 3-6 months; **5.** 7-12 months; **6.** 1-5 years; **7.** more than 5 years
67. Do you currently, regularly contact a body of fresh water?

1. yes- more than once a day; **2.** yes – once daily; **3.** yes – three times a week; **4.** yes – weekly; **5.** yes – monthly; **6.** yes – every other month; **7.** yes – every 6 months; **8.** yes – yearly; **9.** no

68. Number of water contacts per day? (number through 30) ....

69. What is the main source of drinking water for your home?

    1. Piped indoors; **2.** Stand-pipe / tap within plot; **3.** Communal tap; **4.** Borehole; **5.** Protected well; **6.** Unprotected/shallow well/river/dam/lake; **7.** Bowser/tanker; **8.** Other:............................ **9.** no answer

70. What is the main place you go to urinate?

    1. Toilet; **2.** Bush; **3.** Fresh water; **4.** Bush & Fresh water; **5.** Toilet & Fresh water; **6.** Other:................................... **7.** no answer

71. What is the main place you go to defecate?

    1. Toilet; **2.** Bush; **3.** Fresh water; **4.** Bush & Fresh water; **5.** Toilet & Fresh water; **6.** Other:................................... **7.** no answer

72. Which of the following livestock do you live near, if any?

    1. Cattle; **2.** Goats; **3.** Sheep; **4.** Other – specify; **5.** No livestock at home.

73. Which of the following livestock do you have regular contact with, if any?

    1. Cattle; **2.** Goats; **3.** Sheep; **4.** Other – specify; **5.** No regular contact with livestock.

END OF INTERVIEW

Please thank the respondent for sparing time to answer the questions

GUIDING NOTES

15. Schistosomiasis is a disease caused by small worms which infect a person when he gets into contact with infested water, causing bloody urine or stool, abdominal pain, fever and other symptoms

17. Schistosomiasis is caused by small worms which are spread by snails, which are living in freshwaters of rivers, dams and lakes

20. Signs or symptoms of schistosomiasis:

    a) Abdominal pain
    b) Fever
    c) Blood in urine
    d) Blood in stool / faeces
    e) Painful urination
    f) Cough
    g) Headache
    h) Body aches

i) Rash

j) Abdominal swelling (ascites)

23. A healthy person can get infected with schistosomiasis by getting into contact with infested waters with small worms from particular snails in rivers, dams and lakes during routine household chores (washing, bathing, drawing water for home use), recreational (swimming, playing) and income generating activities (fishing, farming, business travel).

25. Schistosomiasis is transmitted from a sick person to healthy person by the former urinating or defecating into waters having particular snails, thereby infecting the snails and after some weeks, the snails release small worms (cercariae) which then penetrate the latter while in the same waters.

29. One can prevent contracting schistosomiasis by avoiding contact with infested water.

32. Problems that schistosomiasis can cause to a person if not treated include:
    a) Abdominal swelling (ascites)
    b) Lesions in genital areas
    c) Paralysis
    d) Seizure
